# *Alpha tubulin* genes from *Leishmania braziliensis:* genomic organization, gene structure and insights on their expression

**DOI:** 10.1186/1471-2164-14-454

**Published:** 2013-07-06

**Authors:** César A Ramírez, José M Requena, Concepción J Puerta

**Affiliations:** 1 Laboratorio de Parasitología Molecular, Departamento de Microbiología, Facultad de Ciencias, Pontificia Universidad Javeriana, Carrera 7 No. 43-82, Edificio 52, Oficina 608, Bogotá, Colombia; 2 Centro de Biología Molecular “Severo Ochoa” (CSIC-UAM), Universidad Autónoma de Madrid, 28049, Madrid, Spain

**Keywords:** *Leishmania braziliensis*, *α-tubulin*, Expression, Untranslated region

## Abstract

**Background:**

Alpha tubulin is a fundamental component of the cytoskeleton which is responsible for cell shape and is involved in cell division, ciliary and flagellar motility and intracellular transport. Alpha tubulin gene expression varies according to the morphological changes suffered by *Leishmania* in its life cycle. However, the objective of studying the mechanisms responsible for the differential expression has resulted to be a difficult task due to the complex genome organization of tubulin genes and to the non-conventional mechanisms of gene regulation operating in *Leishmania*.

**Results:**

We started this work by analyzing the genomic organization of *α-tubulin* genes in the *Leishmania braziliensis* genome database. The genomic organization of *L. braziliensis α-tubulin* genes differs from that existing in the *L. major* and *L. infantum* genomes. Two loci containing *α-tubulin* genes were found in the chromosomes 13 and 29, even though the existence of sequence gaps does not allow knowing the exact number of genes at each locus. Southern blot assays showed that *α-tubulin* locus at chromosome 13 contains at least 8 gene copies, which are tandemly organized with a 2.08-kb repetition unit; the locus at chromosome 29 seems to contain a sole *α-tubulin* gene. In addition, it was found that *L. braziliensis α-tubulin* locus at chromosome 13 contains two types of *α-tubulin* genes differing in their 3′ UTR, each one presumably containing different regulatory motifs. It was also determined that the mRNA expression levels of these genes are controlled by post-transcriptional mechanisms tightly linked to the growth temperature. Moreover, the decrease in the *α-tubulin* mRNA abundance observed when promastigotes were cultured at 35°C was accompanied by parasite morphology alterations, similar to that occurring during the promastigote to amastigote differentiation.

**Conclusions:**

Information found in the genome databases indicates that *α-tubulin* genes have been reorganized in a drastic manner along *Leishmania* speciation. In the *L. braziliensis* genome database, two loci containing *α-tubulin* sequences were found, but only the locus at chromosome 13 contains the prototypic *α-tubulin* genes, which are repeated in a head-to-tail manner. Also, we determined that the levels of *α-tubulin* mRNAs are down-regulated drastically in response to heat shock by a post-transcriptional mechanism which is dependent upon active protein synthesis.

## Background

Alpha tubulin, a highly conserved protein along the eukaryotic evolutionary tree, interacts with *β*-tubulin conforming an *α*/*β*-tubulin heterodimer that comprises the structural subunit of microtubules, the basic building structure of the cytoskeleton, which is responsible for cell shape and is involved in many essential processes, including cell division, ciliary and flagellar motility and intracellular transport [1]. In unicellular microorganisms the cell shape and form are particularly relevant and depend on morphogenetic processes affecting essentially to the internal cytoskeleton. On the other hand, these microorganisms, and particularly the trypanosomatid flagellates, are very adequate models for studying the morphogenetic processes mediated by cytoskeleton reorganizations [2], as many aspects of their biology (infectivity and transmission) depend on these processes.

The *Leishmania* genus comprises at least 20 *Leishmania* species that infect humans, and the spectrum of diseases that they cause can be categorized broadly into three types: self-healing cutaneous leishmaniasis (CL), mucocutaneous leishmaniasis (MCL), and the often fatal visceral leishmaniasis (VL) [3]. Endemic leishmaniasis transmissions have been reported in 98 countries on 5 continents, and around two million new cases of leishmaniasis occur each year [4]. There are two major developmental forms in *Leishmania*, the motile promastigote and the amotile amastigote. Classical studies demonstrated that the promastigote stage synthesizes more tubulin protein than the amastigote stage, and that this biosynthetic change of tubulin was found to correlate with the morphological change of microtubules in leishmanial flagella and cytoskeleton during promastigote-to-amastigote transformation [5]. Studies aimed to uncover the regulation responsible for the differential expression of tubulin genes were initiated shortly after [6], but this has resulted to be a difficult task due to the complex genome organization of tubulin genes [7], in particular, and to the non-conventional mechanisms of gene regulation operating in *Leishmania*[8].

In various *Leishmania* species, the genomic organization of *α-* and *β-tubulin* genes has been analyzed, showing the existence of multiple copies, both arranged in tandem (forming separate clusters of *α-* and *β-tubulin* genes) and dispersed in the genome [7,9,10]. The availability of the genome sequences for several *Leishmania* species [11-13] has allowed resolving questions regarding the genome organization of complex gene families. In a recent work, Jackson and co-workers have carried out a comprehensive study about genomic organization of *β-tubulin* genes in several *Leishmania* species; these authors suggest that the gene organization has evolved to satisfy a need for innovative expression for *β-tubulin* genes [9].

In this work, we studied the organization of *α-tubulin* genes in *Leishmania braziliensis* based on the available, yet incomplete, genome sequence. A comparison with the organization of this gene in *L. infantum* and *L. major* is also provided. The 5´ and 3´ untranslated regions (UTRs) for the different *α-tubulin* genes in *L. braziliensis* have been determined as well as their mRNA expression levels under different conditions.

## Results and discussion

### Genomic organization of *α-tubulin* genes in *L. braziliensis, L. infantum* and *L. major*

**Figure 1:**
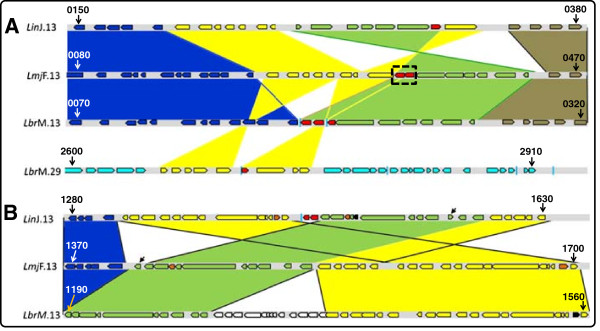
**Genomic organization of *α-tubulin *genes and surrounding regions in three *Leishmania *species.** Panel **A**: comparison among *L. braziliensis* (*Lbr*M) *α-tubulin* loci and syntenic regions in *L. infantum *(*Lin*J) and *L. major* (*Lmj*F) genomes. Panel **B**: comparison among the specific *L. infantum α-tubulin* locus and the syntenic regions of *L. braziliensis* and *L. major*, species in which this locus is absent. Red boxes represent the *α-tubulin* genes. *L. major α-tubulin* locus contains 12 genes (dashed rectangle). Each block of syntenic genes in the different *Leishmania* species, and reorganization events affecting them, is colored differently. The diagonal arrow, point to the LinJ.13.1560 that has an inverted orientation regarding the homologous gene in *L. major* (B). Blue vertical bars denote gaps in sequence. Numbers in bold at the start and end of each gene cluster are the last four digits of GeneDB gene entries.

In the genome of *Leishmania major* (MHOM/IL/81/Friedlin), the first *Leishmania* genome that was sequenced [13], a sole *α-tubulin* locus exists. According to the data available at the GeneDB database [14], the locus is located at chromosome 13 and contains twelve copies (LmjF.13.0280 to LmjF.13.0390) having identical ORFs both in sequence and length (1356 bp), arranged in a head-to-tail tandem organization (Figure 1A). A similar arrangement, containing two copies (LmxM.13.0280 and LmxM.13.0390) separated by a sequence gap, and located also at chromosome 13, was found in the GeneDB database for *Leishmania mexicana* (MHOM/GT/2001/U1103) genome. In contrast, according to the genome database (GeneDB), the *Leishmania infantum* (MCAN/ES/98/LLM-877) genome contains two *α-tubulin* loci, both located at chromosome 13 and separated by a region of 436.6 kb. The more 5´ locus (Figure 1A) contains only a gene copy (LinJ.13.0330, ORF: 1356 bp), whereas the other locus (Figure 1B) has a complete copy (LinJ.13.1460, ORF: 1356 bp) and a truncated one (LinJ.13.1450, ORF: 708 bp). The genome of *Leishmania (Viannia) braziliensis* (MHOM/BR/75/M2904) [13], causative agent of CL and MCL in the New World [15], also contains two loci, one located at chromosome 13 and the other at chromosome 29. The locus at chromosome 13 (Figure 1A) is composed by two complete copies (LbrM.13.0190 and LbrM.13.0200, ORFs: 1356 bp) and an *α-tubulin-*like gene (LbrM.13.0210, ORF: 702 bp). The locus at chromosome 29 (Figure 1A) contains a sole copy consisting of a truncated form of the *α-tubulin* gene (LbrM.29.2700, ORF: 780 bp).

Comparison of the genomic region around the *α-tubulin* loci in *L. major, L. infantum* and *L. braziliensis* revealed that the chromosomal regions surrounding the *α-tubulin* genes at chromosome 13 have experienced remarkable reorganizations in these three *Leishmania* species, and the *α-tubulin* locus itself appears as the target of DNA deletion and inversion events (Figure 1A). Thus, between *L. major* and *L. infantum* an inversion including the LmjF.13.0270 gene (colored in yellow in Figure 1A), the *α-tubulin* locus *(*LmjF.13.0280 toLmjF.13.0390, colored in red in Figure 1A) and the LmjF.13.400 to LmjF.13.450 genes (colored in green in Figure 1A) took place. As a result of this event, the ORF polarity of the LinJ.13.0270 to LinJ.13.0320, *α-tubulin* (LinJ.13.0330) and LinJ.13.0340 genes was changed with respect to the equivalent genes of *L. major*. In *L. braziliensis*, regarding the *L. major* chromosomal organization, a region spanning from the LmjF.0200 to the LmjF.0270 gene, corresponding to a string of 8 genes encoding for 7 hypothetical proteins and one putative class 3 lipase (colored in yellow in Figure 1A), was deleted*.* These deleted genes are present at chromosome 29 (LbrM.29.2650 to LbrM.29.2730, colored in yellow in Figure 1A) in the *L. braziliensis* genome; this reorganization that seems the result of two transposition events dragged a region of the *α-tubulin* locus, generating the LbrM.29.2700 gene (colored in red in Figure 1A), which contains a truncated sequence of the prototypical *α-tubulin* gene (see below).

The other *α-tubulin* locus at *L. infantum* chromosome 13, comprising as mentioned above a partial and a complete copy of *α-tubulin* coding sequence (Figure 1B), is found in a region that also has experienced clear reorganizations in these three *Leishmania* species. Thus, the regions flanking the *α-tubulin* genes LinJ.13.1450 and LinJ.13.1460 in *L. infantum* chromosome have experienced a substantial reorganization regarding the equivalent chromosomal region in *L. major*, and again the *α-tubulin* locus (absent in *L. major*) appeared as the center of the reorganization events (Figure 1B). Another minor transposition event, regarding the LinJ.13.1560 gene (signaled by a diagonal arrow in Figure 1B) took place after separation of these two *Leishmania* species. This chromosomal region in *L. braziliensis* (Figure 1B) is very similar to the *L. major* homologous region (*α-tubulin* genes are also absent), excepting an insertion comprising the LbrM.13.1250 to LbrM.13.1370 gene block (Figure 1B). The LbrM.13.1250-LbrM.13.1370 orthologous are located on chromosome 34 in both *L. major* and *L. infantum* (data not shown).

In summary, these analyses pointed out that the *α-tubulin* gene loci have been reorganized in a drastic manner along *Leishmania* speciation. However, this fact contrasts with the chromosomal organization of the three *β-tubulin* loci that is largely syntenic in *L. major, L. infantum* and *L. braziliensis*[9].

Nevertheless, it cannot be excluded the possibility that differences in the chromosomal distribution and organization of *α-tubulin* genes among *L. major*, *L. infantum* and *L. braziliensis* are the result of artifacts generated during assembling of contigs into chromosomal scaffolds. The *L. infantum* and *L. braziliensis* genome sequences were produced by whole-genome shotgun sequencing to five- and six-fold coverage, respectively [12]. The resulting assemblies of *L. infantum* and *L. braziliensis* yielded 470 and 1,031 contigs, respectively. Finally, chromosomal scaffolds were produced by aligning contigs against the reference *L. major* sequence [13]. However, sequence gaps remain to be determined in the genome sequences for both *L. infantum* and *L. braziliensis* (according to the GeneDB database). Remarkably, the *α-tubulin* locus at chromosome 29 in *L. braziliensis* (Figure 1A) and one of the *α-tubulin* loci at chromosome 13 of *L. infantum* (Figure 1B) are located at the end of contigs (next to sequence gaps).

### Copy number determination in the *L. braziliensis α-tubulin* loci

**Figure 2:**
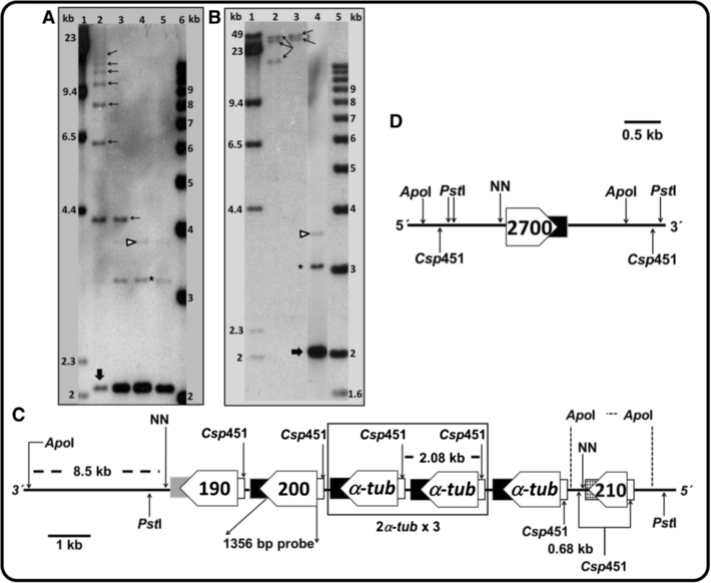
**Genomic organization of *L. braziliensis α-tubulin *genes.** Southern blot of promastigote DNA hybridized with the *α-tubulin* ORF. **(A)** Two μg of *L. braziliensis* DNA were partially digested with *Csp*451 for 2 min (lane 2); 5 min (lane 3) and 15 min (lane 4); lane 5 contains 1 μg of totally digested DNA. Thin arrows in panel A point to the ladder bands; a thick arrow (in panels A and B) points to the repeated unit; the arrow head and the asterisk (in panels A and B) mark additional hybridization bands. **(B)** Southern blot corresponding to 3 μg of *L. braziliensis* DNA digested with either *Pst*I (lane 2) or *Apo*I (lane 3); 5 min of film exposure. Lane 4 shows a Southern blot of 3 μg of *Csp*451-digested DNA; 20 min of film exposure. Thin arrows point to hybridization bands. Molecular weight markers: lanes 1, a mixture of undigested and *Hin*dIII-digested DNA from λ phage; lanes 6, 1-Kb plus marker (Invitrogen California, USA). The position of the DNA markers in the blot was revealed by including digoxigenin-labeled markers in the hybridization mixture. As probe, the *L. braziliensis α-tubulin* ORF was used. **(C)** Hypothetical map for the *α-tubulin* locus in *L. braziliensis* chromosome 13, as deduced from Southern blot analyses and genomic sequence at GeneDB database **(D)**. Map for *α-tubulin* locus in *L. braziliensis* chromosome 29, as deduced from genomic sequence at GeneDB database. Pentagonal shaped boxes represent *α-tubulin* genes, numbers inside each box are verb GeneDB entries (LbrM.13.0190, LbrM.13.0200, LbrM.13.0210 and LbrM.29.2700); filled boxes at the end of each gene represent the 3´ UTR; and rectangles located at the 5´ end of each gene, the 5´ UTR.

As shown in Figure 1, the genomic regions containing the two *α-tubulin* loci in chromosomes 13 and 29 of *L. braziliensis* has not been sequenced in full; there are sequence gaps that do not permit deducing the number of genes present at each locus. Moreover, tandem gene arrays are among the most challenging to resolve correctly using current genome sequencing technologies, since repetitive sequence reads tend to collapse into a single contig when no variation exists to distinguish them. Previous studies in *L. major*[16] and *L. enriettii*[10] showed the existence of tandem arrangements of *α-tubulin* genes; in fact, the *L. major* genome database includes twelve gene copies tandemly arranged at chromosome 13. In order to determine the number of genes and their organization in the two *α-tubulin* loci of *L. braziliensis,* Southern blots containing DNA digested with selected restriction enzymes were hybridized with a probe derived from the *α-tubulin* coding region (Figure 2). Southern blot analysis of genomic DNA, partially digested with the *Csp*451 restriction enzyme, evidenced the typical ladder of tandemly repeated genes (Figure 2A). At least 8 copies of the repeated unit (2.08 kb in length) were observed. According to the restriction maps of the two *α-tubulin* loci (Figure 2B, 2C and 2D), this tandem should be present in the locus at chromosome 13, whereas the locus at chromosome 29 would contain a sole *α-tubulin* gene (see the hybridization band pointed by the triangle in Figure 2A, and 2B). Recently, Rogers et al. [11], analyzing Illumina high-throughput sequencing data, inferred that *L. braziliensis* genome should contain 15 *α-tubulin* copies (haploid content). Tandemly repetition of genes has been proposed as a direct mechanism for increasing the transcript abundance of highly expressed proteins [8], and it is known that *α*-tubulin is an abundant protein in *Leishmania*[2].

When *L. braziliensis* total DNA was digested with restriction enzymes lacking cut sites within the *α-tubulin* loci (*Pst*I and *Apo*I), and the hybridization pattern was analyzed by Southern blotting, a conspicuous result was obtained (Figure 2B). The presence of three (*Pst*I) and two (*Apo*I) large bands was unexpected. An explanation to this finding may be either the existence of allelic sequence polymorphisms in the regions surrounding the *α-tubulin* locus at chromosome 13 or different copy numbers of *α-tubulin* genes in each homologous chromosome. Indeed, similar observations have been described for *L. braziliensis* and other trypanosomatids regarding *HSP70, KMP-11 and histone H2A* gene loci [17-19]. Moreover, the presence of three hybridization bands in the lane containing *Pst*I digested-DNA would be indicating that *L. braziliensis* has three homologous for chromosome 13, a very plausible possibility taking into account recent studies showing that *L. braziliensis* genome is essentially triploid [11].

### Sequence analysis of the *α-tubulins* in *L. braziliensis*

**Figure 3:**
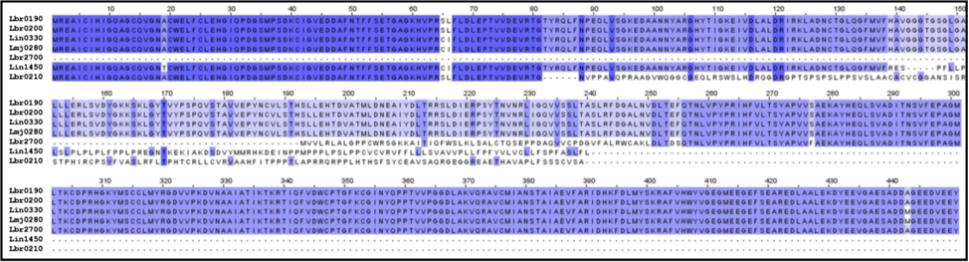
**Comparison of *α*-tubulin sequences among *Leishmania *species.** Multiple sequence alignment of LbrM.13.0190 (Lbr0190), LbrM.13.0200 (Lbr0200), LinJ.13.0330 (Lin0330), LmjF.13.0280 (Lbr0280), LbrM.29.2700 (Lbr2700), LinJ.13.1450 (Lin1450) and LbrM.13.0210 (Lbr0210) deduced amino acid sequences, constructed using Clustal-W and Jalview tools.

The genomic analysis described above indicated that the *L. braziliensis α-tubulin* genes are encoding for three different amino acids polypeptides: the prototypical *α*-tubulin (encoded by genes LbrM.13.0190 and LbrM.13.0200) and two *α-tubulin* variants, dubbed *α-tubulin*-A (LbrM.13.0210) and *α-tubulin*-B (LbrM.29.2700) (Figure 3). The prototypical *α-tubulin* is a 451 amino acid protein with a molecular weight of 49.7 kDa and an isoelectric point of 4.9. In contrast, the *α*-tubulin-A is a shorter sequence with 233 amino acids, molecular weight of 24.9 kDa and isoelectric point of 6.9. Similarly, the *α*-tubulin-B sequence contains 259 amino acids, has a molecular weight of 28.8 kDa and an isoelectric point of 5.0. As shown in Figure 3, the *α*-tubulin-A is identical to the *α*-tubulin in the 81 N-terminal amino acids, but they are absolutely divergent in the rest of the sequence. An intriguing finding was the observation of a remarkable sequence identity (100%) between the divergent region of gene LbrM.13.0210 and a genomic region of chromosome 28, located at the intercoding region of genes LbrM.28.2580 and LbrM.28.2590 (see Additional file 1).

In contrast, *α*-tubulin-B sequence, excluding the 58 N-terminal amino acids, was found to be identical to the C-terminal half of the prototypical *α*-tubulin. Also, this *α*-tubulin protein variant may have an extended sequence at the N-terminal region, since the LbrM.29.2700 annotated gene is close to a gap in the genomic sequence of the *L. braziliensis* genome database (Figure 1A).

As mentioned above, if assembly artifacts exist in the *L. braziliensis* genome database, the real existence of the *α*-tubulin variants encoded by genes LbrM.13.0210 and LbrM.29.2700 may be questioned. To elucidate this, we have searched in the literature for experimental evidence of these genes or their products. The sole reference found is their presence in the gene lists generated by Rogers and co-workers [11] when analyzed the copy number of genes along the genome of *L. braziliensis*. However, this is not a demonstration of the real existence of these genes as the copy number was determined by mapping Illumina reads to the reference genome [12], but it was not analyzed whether the reads map homogenously along the gene ORF. On the other hand, none of these genes have homologs in the *L. braziliensis* MHOM/BR/75/M2903 strain [20], in other *Leishmania* species, even though several genomes have been sequenced (*L. panamensis*, *L. major*, *L. infantum*, *L. mexicana*, *L. donovani* and *L. tarentolae*), or in related trypanosomatids (*Trypanosoma brucei* and *T. cruzi*). Finally, an additional finding pointing to a possible assembly error in gene LbrM.13.0210 is the fact that the region conserved with α-tubulin would extend further (35 amino acids) if the nucleotide at position 245 is deleted (data not shown).

### The *L. braziliensis α-tubulin* locus at chromosome 13 contains two types of *α-tubulin* genes differing in their 3′ UTR

**Figure 4:**
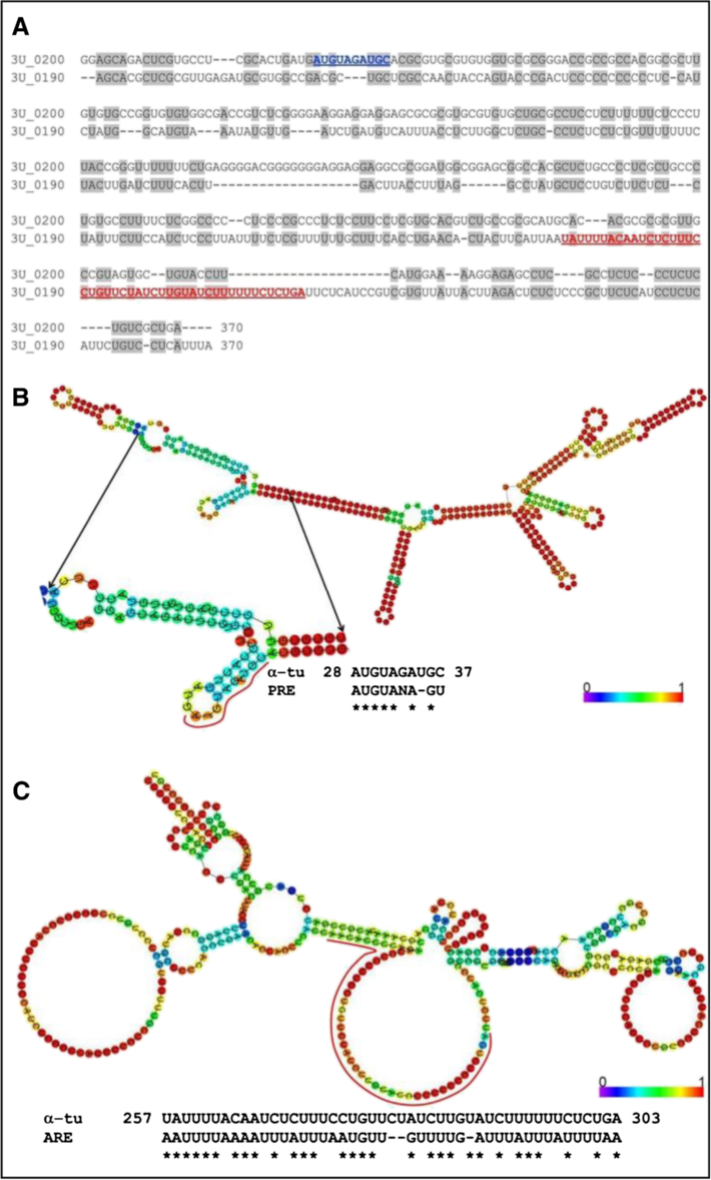
**Analysis of the 3´ UTR of *α-tubulin *genes from *Leishmania braziliensis*. (A)** Alignment between the 3´ UTR-I (LbrM.13.0200) and the 3´ UTR-II (LbrM.13.0190). **(B)** PRE element (highlighted in blue in A) identified in the 3´ UTR-I. **(C)** ARE element (highlighted in red in A) observed in the 3´ UTR-II. The color scale in B and C denotes the structure probability from blue (0) to red (1), according to [44].

Given that *Leishmania* genes are transcribed into polycistronic RNA precursors that need to be further processed into individual mRNAs by *trans*-splicing and polyadenylation, post-transcriptional regulation represents the main level of controlling gene expression in these parasites [8,21]. Consequently, regulatory sequences located in the 5´ and 3´ untranslated region (UTR) are involved in the modulation of mRNA processing, mRNA stabilization/destabilization, mRNA half-life, or translation efficiency [8,21]. To determine the UTRs for *L. braziliensis α-tubulin* gene, RT-PCR reactions using specific oligonucleotides were performed. Thus, it was possible to determine the 5´ UTR region of the *α*-*tubulin* genes LbrM.13.0190 and LbrM.13.0200, which was 69 nucleotides in length and identical for both genes. This sequence was deposited in the GenBank database under the accession number FJ750454, and it was incorporated to the GeneDB database [22]. Regarding the 3´ UTR, by RT-PCR we determined that this region for LbrM.13.0200 (and probably for other genes present in the locus, see below) is 369-nucleotide in length (GenBank accession number FJ750455); henceforth this sequence will be referred as 3´ UTR-I. On the other hand, the 3´ UTR of the last gene of the cluster (i.e. LbrM.13.0190), referred here as 3´UTR-II, was found to be highly divergent (Figure 4A). Taking into account that the *L. braziliensis α-tubulin* locus at chromosome 13 should be composed by 8–15 copies of the prototypic *α-tubulin* gene, but they collapsed to only two (LbrM.13.0190 and LbrM.13.0200) during assembly process, it is reasonable concluding that the 3´ UTR for the rest of genes would be identical or near-identical to the 3´ UTR-I. Similar situation has been reported in *β tubulin* array from *L. major*[9], and *HSP70* genes [17] in which the last gene of the tandem bears a 3´ UTR different to that found in the rest of genes. The distinctive genomic environment provided by each 3´ UTR type (I and II) might obey to a need of providing specific expression profiles that ensure the availability of the *α*-tubulin protein under different conditions as occurs with the HSP70 protein [23]. Remarkably, bioinformatics analysis of these two sequences showed that whereas the 3´ UTR-I (LbrM.13.0200 gene) has a PRE motif (Figure 4B), which has been implicated in the promastigote-specific expression of several genes [24], the 3´ UTR-II (LbrM.13.0190 gene) has an ARE motif (Figure 4C), which is recognized as an mRNA instability element [25].

### The mRNA levels for *L. braziliensis α*-tubulin are controlled post-transcriptionally by mechanisms sensitive to changes in environmental temperature

Analysis of *α-tubulin* gene expression in *L. braziliensis* has not been addressed to date. Early studies in *Leishmania enriettii* demonstrated that in agreement with the greater tubulin content in the promastigote stage, the levels of *α-* and *β-tubulin* mRNAs in the promastigotes were significantly higher than those found in amastigotes [26]. In *L. major*, using microarray hybridizations, it was found that *α-tubulin* mRNAs are 6.3-fold more abundant in promastigotes than in amastigotes [27]. However, in *L. mexicana*, similar amounts of *tubulin* mRNAs were found in both promastigotes and amastigotes [28,29]. In the related trypanosomatid *T. cruzi*, the regulation of *tubulin* genes has been studied in some extent [30,31]. In this parasite, lower levels of the *α-* and *β-tubulin* transcripts are found in amastigotes and trypomastigotes compared to epimastigotes. Experimental evidence suggests *tubulin* mRNA half-lives are negatively affected by the amount of free, unpolimerized tubulin proteins. The regulatory mechanism would operate through the recognition of sequence elements located at the beginning of the coding region and in the 3´ UTR of the *tubulin* genes [31].

**Figure 5:**
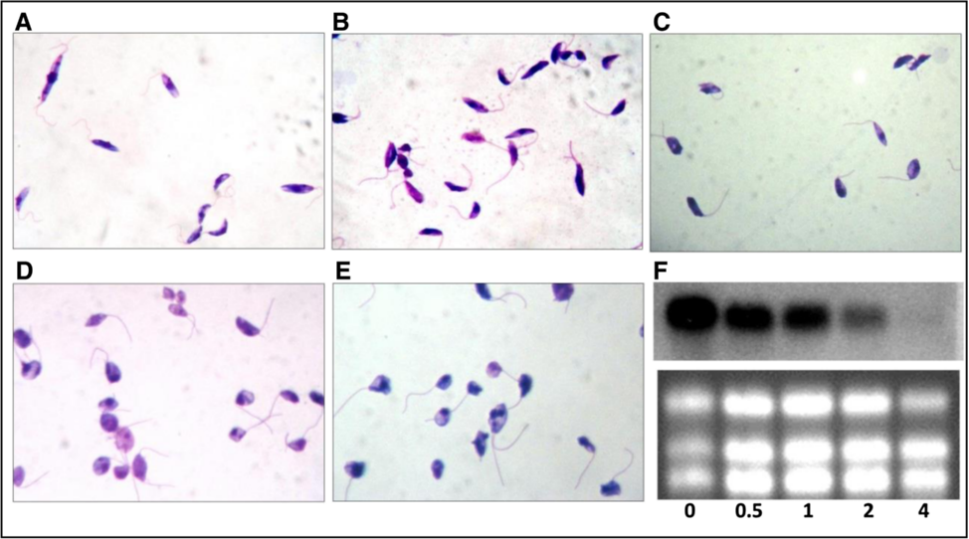
***Leishmania braziliensis *morphology and *α-tubulin *RNA accumulation after heat shock treatment.** Promastigotes cultures were incubated at 26 **(A)** or 35°C with 5% CO_2_ during 30 min **(B)**, 1 hour **(C)**, 2 hours **(D)** and 4 hours **(E)**. Total RNA was extracted from each time point, and RNA aliquots of 8 μg each were analyzed by Northern blotting **(F)**. The blots were hybridized with the *α-tubulin* ORF probe and ethidium bromide rRNA staining was used as normalizing signal.

Temperature shifts trigger differential gene expression and stage transformation in *Leishmania*[32]. Thus, we first examined the effect of heat shock treatment on the *L. braziliensis α-tubulin* mRNA stability. For this purpose, parasites were cultured at 35°C during several periods of time, determining the parasite morphology and *α-tubulin* mRNA abundance at each time culture-point. As shown in Figure 5, as incubation time at 35°C was increased, clear alterations in the parasite morphology were observed; parasites turned into rounded shape forms. Interestingly, a concomitant decrease in the *α-tubulin* mRNA abundance was also observed, suggesting a down-regulation of *α-tubulin* mRNAs levels during adaptation of the parasite to the mammalian host temperature.

**Figure 6:**
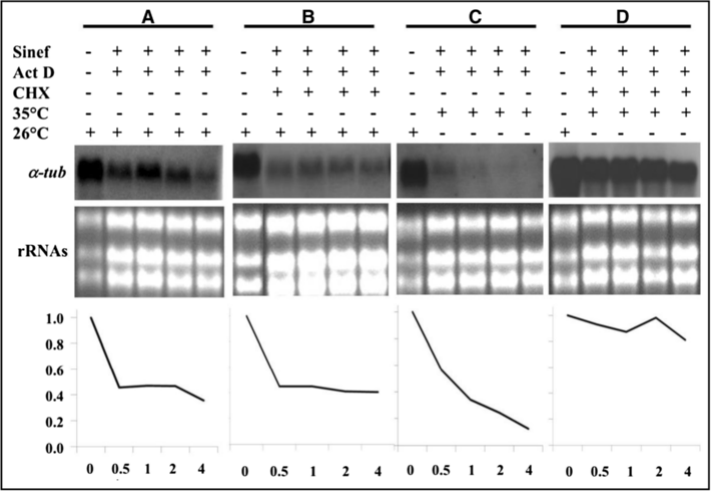
**Heat shock treatment and protein synthesis inhibition effect on the stability of *α-tubulin *transcripts.** For total transcription inhibition, promastigotes cultured at 26°C were incubated with 10 μg/ml sinefungin (Sinef) five minutes previously to the addition of 10 μg/ml actinomycin D (Act D). Afterwards, cultures were incubated either at 26°C **(A)** or 35°C with 5% CO_2_**(C)** for 0, 0.5, 1, 2, and 4 hours. For protein synthesis inhibition, 20 μg/ml of cycloheximide (CHX) was used in promastigotes cultured either at 26°C **(B)** or 35°C with 5% CO_2_**(D)** for 0, 0.5, 1, 2, and 4 hours. Finally, total RNA was extracted, and 8 μg from each sample were analyzed by Northern blotting. The blots were hybridized with *α-tubulin* ORF probe. Before transferring, gels were stained with ethidium bromide and photographed; rRNA staining was used as normalizing signal for densitometric measurement. For each series, densitrometric graphs (bottom panels) were standardized against the RNA signal found at time 0 (26°C), which was arbitrarily set as 1.0.

In order to obtain clues about the regulatory mechanisms regulating mRNA stability of the *L. braziliensis α-tubulin* genes, we used inhibitors of transcription (actinomycin D), *trans*-splicing (sinefungin) and protein synthesis (cycloheximide). The effects of these drugs were analyzed at both 26°C and 35°C for several periods of time (Figure 6). As expected, inhibition of transcription and mRNA processing at 26°C, led to a continuous decrease in the *α-tubulin* mRNAs (Figure 6, panel A). The decrease was more dramatic in parasites incubated at 35°C (Figure 6, panel C), suggesting the existence of a destabilizing mechanism operating at this temperature. A support for the existence of such a specific mechanism came from the observation that protein synthesis inhibition of *L. braziliensis* promastigotes at 35°C (Figure 6, panel D) but not at 26°C (Figure 6, panel B), led to an increase of the *α-tubulin* mRNA half-lives. Moreover, protein synthesis inhibition at 26°C (Figure 6, panel B) seems to have no effect on the decay rate of the *α-tubulin* mRNAs (Figure 6, panel A). Thus, it can be hypothesized the existence of a labile protein factor, induced during promastigote to amastigote differentiation, that is responsible of decreasing the *α-tubulin* mRNA half-lives in the mammalian stage of *L. braziliensis*. This finding would be in agreement with the fact, known since the 1980 decade, that tubulins are more abundant proteins in the promastigote stage than in the amastigote one [5,28,29].

Gene expression in trypanosomatids is controlled by post-transcriptional mechanisms relying on the modulation of mRNA processing, mRNA stabilization/destabilization, mRNA half-life, translation efficiency or post-translational modifications [8,21]. Accordingly, a variety of elements in the untranslated regions (UTR) have been shown to regulate these events [33-35]. Now, in this study, the genomic organization and the structure of 5′ and 3′ UTRs for *α-tubulin* genes have been determined. Next step will be to investigate the involvement of the motifs contained in the 3′ UTR-I and -II sequences in the regulatory mechanisms controlling tubulin expression in *L. braziliensis*.

## Conclusions

The analysis of the genomic organization and genome distribution of *α-tubulin* genes in several *Leishmania* species suggests that these genes have been reorganized in a drastic manner along *Leishmania* speciation. Nevertheless, the existence of a gene cluster, consisting of several copies of tandemly arranged *α-tubulin* genes, was found to be a common feature in the different *Leishmania* species. In the *L. braziliensis* tandem array, two types of *α-tubulin* genes differing only in their 3´ UTR were characterized: most of the *α-tubulin* gene copies have an identical 3´ UTR (named 3´ UTR-I), whereas the last copy of the tandem possesses a different sequence (named 3´ UTR-II). Finally, we demonstrated that mRNA expression of *L. braziliensis α-tubulin* genes is controlled by a temperature-dependent post-transcriptional mechanism.

## Methods

### Parasite cultures and nucleic acids extraction

Promastigotes of *L. braziliensis* MHOM/BR/75/M2904 were cultured *in vitro* either at 26°C in Schneider’s insect medium (Sigma Aldrich**,** Inc., St. Louis, USA)supplemented with 20% heat-inactivated fetal calf serum (Eurobio, Inc., Les Ulis, France), and 0.1 μg/mL of 6-biopterin (Sigma Aldrich,Inc., St. Louis, USA) or at 35°C with 5% CO_2_ by several time periods**.** Total DNA and RNA from parasite cells were isolated using the phenol-chloroform-isoamilic alcohol method [17], and the TRIzol method (Invitrogen, California, USA) according to manufacturer instructions, respectively.

### Oligonucleotides

All oligonucleotides were synthesized by IDT, Inc. (Miami, USA). The *L. braziliensis α-tubulin* coding region was amplified from genomic DNA by PCR with Lb-Tub-F (5´ GGATCCATGCGTGAGGCTATCTGC 3´) and Lb-Tub-R (5´ CTGCAGCTAGTACTCCTCGACGTCCT 3´) primers, which contain the *Bam*HI and *Pst*I restriction sites (underlined in the sequence), respectively. Amplification of the UTRs was performed from poly-T primed-cDNA using the following primers: LbSL (5´ CGCTATATAAGTATCAGTTTC 3´) and Lb*α*T-106 (5´ GTGAGGCTATCTGCATTCA 3´) for the 5´ UTR and Lb*α*t1312 (5´ TCGTGCACTGGTACGTTG 3´) and poly-T *Eco*RI (5´ CGGAATTCTTTTTTTTTTTTTTTTTTT 3´), for the 3´ UTR.

### Cloning sequences and *in silico* analyses

First-strand cDNA synthesis was carried out from *L. braziliensis* total RNA using an oligo-dT primer and the Transcriptor first strand cDNA synthesis kit (Roche, Inc., Mannheim, Germany). The following PCR mix reaction was used for all amplifications in a final volume of 20 μl: 1× reaction buffer (10 mM Tris-HCl pH 9.0, 50 mM KCl, 0.1% Triton X-100), 1.5 mM MgCl_2_, 0.4 mM of dNTP mix, 0.5 μM of each primer, 1 M betain, 0.06 units per μl of expand high fidelity enzyme (Roche, Inc., Mannheim, Germany) and 15 ng/μl DNA or 1 μl of cDNA product. An MJ Research PTC-100 DNA thermocycler was used for the reaction with the following amplification profile: 95°C/5 min (initial denaturation), 35 cycles at 92°C/0.5 min, annealing at 52-58°C/0.5 min and extension for 72°C/1 min, with a final incubation at 72°C for 10 min. All the amplified fragments were resolved in agarose gels and visualized under UV exposure after ethidium bromide staining. RT-PCR products were excised from gels, purified using Wizard® SV Gel and PCR Clean-Up System (Promega, Inc., Madison, WI, USA) and cloned into the pCR®II (Invitrogen, California, USA) or pGEM®-T Easy plasmids (Promega, Inc., Madison, WI, USA). The following clones were obtained and submitted to the Addgene web site [36] pTOLb*α*tub-B (Addgene ID, 42937), containing the *α-tubulin* ORF, pLb5*α*Tub-D (Addgene ID, 42938), the 5´ UTR and pLb3*α*Tub-C6 (Addgene ID, 42939), the 3´ UTR. The sequences from each plasmid insert were determined using the Big Dye Terminators v3.1 kit (Applied Biosystem, California, USA) by automatic sequencing at the Servicio de Genómica (Parque Científico de Madrid, Universidad Autónoma de Madrid). In order to deduce the *α-tubulin* mRNA UTRs from other *Leishmania* species, LALIGN [37], and ClustalW analyses [38] were carried out. Gene comparison for investigating the constitution of *α-tubulin* variants was performed by BLAST/N analysis [20]. Data from TriTrypDB [39] were retrieved for studying the *α-tubulin* loci synteny and comparison among the three *Leishmania* species was carried out by BLAST/N analysis.

### Southern blot analyses

With the aim of choosing endonucleases cutting at the boundaries of the *α-tubulin* locus (*Pst*I and *Apo*I) or inside it (*Csp*451), sequences of *α-tubulin* loci from the three *Leishmania* species were retrieved from GeneDB database [40] and used in an *in silico* restriction analysis through NEBcutter V2.0 tools [41]. DNA from promastigotes was totally or partially digested with the selected restriction enzymes according to the manufacturer specifications (Promega, Inc., Madison, WI, USA). Resulting fragments were electrophoresed during sixteen to eighteen hours at 50 volts on 25 cm low electroendosmosis agarose gel at 0.8% (Ambion, Inc., Texas, USA), transferred to a nylon membrane (Roche, Inc., Mannheim Germany) by the saline-sodium citrate (SSC) buffer method [42] and hybridized with the *α-tubulin* ORF as probe. For probe synthesis, the *L. braziliensis α-tubulin* gene was released from clone pTOLb *α* tub-B by *Bam*HI/*Pst*I cutting, excised from gel, purified using Wizard® SV Gel and PCR Clean-Up System (Promega, Inc., Madison, WI, USA), and finally labeled with digoxigenin by randomly primed synthesis using the DIG High Prime DNA Labeling kit (Roche, Inc., Mannheim, Germany). Hybridizations were performed using the Detection Starter kit II (Roche, Inc., Mannheim, Germany) according to manufacturer's instructions. Afterwards, membranes were washed twice under the following conditions: 2× sodium citrate solution (SSC) and 0.5% (p/v) sodium dodecyl sulfate (SDS) at room temperature for 5 min, 0.5 × SSC, 0.1% SDS solution for 15 min at 65°C and 0.1 × SSC, 0.1% SDS solution for 15 min at 65°C, under constant shaking. Finally, after the immunological detection according to the above mentioned kit recommendations, membranes were exposed on Curix RP2 plus medical X-Ray film (AGFA, Mortsel, Belgium).

### Northern blot analyses

To investigate the *α-tubulin* expression levels, *L. braziliensis* promastigote cultures were treated as follows: maturation of mRNA were inhibited by addition of 10 μg/ml sinefungin to the *L*. *braziliensis* promastigote cultures at logarithmic phase (5–9 × 10^6^ promastigotes per ml), synthesis of RNA were inhibited by addition of 10 μg/ml actinomycin D five min later [43]. Protein synthesis inhibition was achieved by adding 10 μg/ml of cycloheximide on promastigote cultures simultaneously treated with sinefungin and actinomycin D. Parasites were treated at 26 or 35°C with 5% CO_2_, with or without inhibitors for several time periods (0.5, 1, 2 and 4 hours). Afterwards, parasites were harvested for RNA extraction and 8 μg of total RNA per line were separated on 1.5% (w/v) low electroendosmosis agarose/MOPS/formaldehyde gels (Ambion, Inc., Texas, USA) and transferred to nylon membranes (Roche, Inc., Mannheim, Germany). Probes, hybridization and immunological detection were performed as previously mentioned.

## Competing interests

The authors declare that they have no competing interests.

## Authors’ contributions

CAR, JMR, and CJP conceived and designed the experiments. CAR performed the experiments. CAR, JMR, and CJP analyzed the data. CAR, JMR, and CJP wrote the paper. All authors revised and approved the final version of the manuscript.

## Supplementary Material

Additional file 1: Figure S1 (A) The 3′ end of LbrM.13.0210 gene is derived from chromosome 28. In the intercoding region of LbrM.28.2580 and LbrM.28.2590 entries at chromosome 28 from *L. braziliensis* there is a 457 nucleotide sequence that is identical to the final 355 nucleotides of the LbrM.13.0210 ORF and the first 102 downstream nucleotides. Boxes in red comprise ORFs. (B) Alignment of the LbrM.13.0210 entry with the intergenic region between the LbrM.28.2580 and the LbrM.28.2590 entries (28IGR). A 100% of identity in 457 nt overlapped, showed in red, was found. The ATG and stop codons of the LbrM.13.0210 entry are indicated in blue. Click here for file
