# Supplementary material for: Alpha tubulin genes from Leishmania braziliensis: genomic organization, gene structure and insights on their expression
Source: BMC Genomics. 2013 Jul 6;14:454. doi: 10.1186/1471-2164-14-454 (PMC3708823; doi:10.1186/1471-2164-14-454)
Supplement: Additional file 1: Figure S1 — (A) The 3′ end of LbrM.13.0210 gene is derived from chromosome 28. In the intercoding region of LbrM.28.2580 and LbrM.28.2590 entries at chromosome 28 from L. braziliensis there is a 457 nucleotide sequence that is identical to the final 355 nucleotides of the LbrM.13.0210 ORF and the first 102 downstream nucleotides. Boxes in red comprise ORFs. (B) Alignment of the LbrM.13.0210 entry with the intergenic region between the LbrM.28.2580 and the LbrM.28.2590 entries (28IGR). A 100% of identity in 457 nt overlapped, showed in red, was found. The ATG and stop codons of the LbrM.13.0210 entry are indicated in blue. [file 1471-2164-14-454-S1.pdf]

A

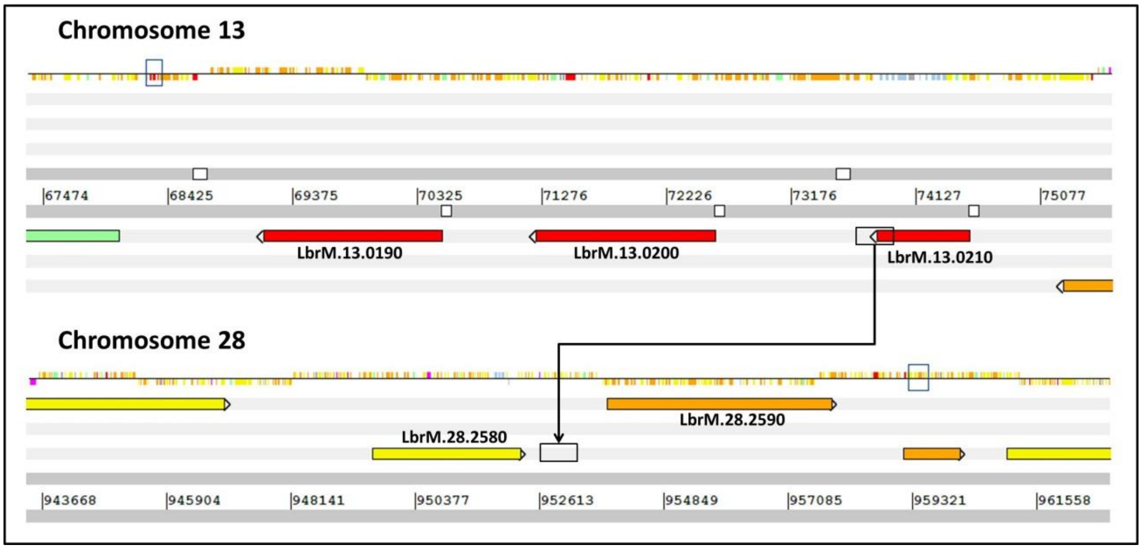

B

```
28IGR  ----------------------------------------------------TTCCGTTGGTAGAGGGGAGCTAAAGGGAGTACGAGTGCCACCTCTTTACTTCCCCTCTGCCTCCTCCC
                                                           :::     :::     ::     ::: :::    ::: : :   ::  : ::    ::::   :
at0210 ATGCGTGAGGCTATCTGCATTCACATCGGCCAGGCCGGCTGCCAGGTCGGTAACGCGTGCTGGGAGCTCTTCTGCCTTGAGCACGGCATCCAGCCCG--ACGGCTCCATGCCCTCTGACA

28IGR  CTAGCATTGTTGCTGCTGCTGCTGCTGCTATGCTTTGTGCCTGCGCATCTATGCGGGAATAGATGAGTGCGTGCTTACTGG-CATAGGCGAGGATTCGTGTTTCGTTACTGACGCTA
          :::::: :: ::  : ::  ::   : : :  : :: : :  :   : :   :: ::   ::     : :  :::  ::   : : : :   ::  :     ::::    ::::
at0210 AGTGCATTGGTGTTGAGGATGACGCG--TTCAACACCTTCTTCTCGGAGACCGGAGCTGGCA-AGCACGTTCCTCGCTCCCTCTTCCTGGACCTCGAGCCCACGGTCGTG---GACG---

28IGR  CCACGATCGATAGGGGGAGGGACGGGGGAGGGGGTAAAACGCGTTTA-CTTTTGTATGTGCACGTCTGTGTAGAGGAGCGTGGACCTTCATTCAC-CTGCACTTCC--CCACGACACCGC
         : :  ::    ::: : : :: :   ::  : : :: : ::   : ::  ::: :: : :: :    :    :  :: :    ::  :: :   : :: :::    :   : : :: : ::
at0210 --AGGTGCGCACTGGGAACGTACCGCC-AGCTGTTCAACCCCGAGCAGCTGGTGTCTG-GCAAGGAGGATGCGGCGAACAACTACGCTCGTGGTCACTACACGATCGGCAAGGAGATCGT

28IGR  ---CCCACCAGCCCCTCTCCCTCCCTCCCTCCCTCCGTGTCTCTTGCGGCATGTGCGTGCGTCTGTGGTGGTGCCAATTCCATCTCCCGAAGTACTCCGCACATCCGGTGTCCCTCTTAC
          :::::::::::::::::::::::::::::::::::::::::::::::::::::::::::::::::::::::::::::::::::::::::::::::::::::::::::::::::::
at0210 GGACCCACCAGCCCCTCTCCCTCCCTCCCTCCCTCCGTGTCTCTTGCGGCATGTGCGTGCGTCTGTGGTGGTGCCAATTCCATCTCCCGAAGTACTCCGCACATCCGGTGTCCCTCTTAC

28IGR  TTTGTTGCTTCCTTACGATTCTTAACCCCTCACACTTGTAGGCTGTTGTGCGTGCGTGTGGCTGCCCACTTCATCACGCCTCCACCAACTCTCGCACCGCGCCGCCAAAGGCCGCCGTTG
       ::::::::::::::::::::::::::::::::::::::::::::::::::::::::::::::::::::::::::::::::::::::::::::::::::::::::::::::::::::::
at0210 TTTGTTGCTTCCTTACGATTCTTAACCCCTCACACTTGTAGGCTGTTGTGCGTGCGTGTGGCTGCCCACTTCATCACGCCTCCACCAACTCTCGCACCGCGCCGCCAAAGGCCGCCGTTG

28IGR  CACACGCACTCCTTTTCGTATTGTGAGGCGGTCAGTGCACAGAGGGGGGAGGGAGGAAGAGAGGCAGAGACACATGCTGTCGCACCCTTATTCTCCTCGTCATGCGTTTCGGCCTAGCCA
       ::::::::::::::::::::::::::::::::::::::::::::::::::::::::::::::::::::::::::::::::::::::::::::::::::::::::::::::::::::::
at0210 CACACGCACTCCTTTTCGTATTGTGAGGCGGTCAGTGCACAGAGGGGGGAGGGAGGAAGAGAGGCAGAGACACATGCTGTCGCACCCTTATTCTCCTCGTCATGCGTTTCGGCCTAGCCA

28IGR  CACCTCACCGGCATACGAATAAACTACGCTGGGGCGCGTTCATCTTTGTGTCGATCTCTGTTGTGTTCTTTCCCCTTCATAGCCGCACACACACACACAC-----------------CT
       ::::::::::::::::::::::::::::::::::::::::::::::::::::::::::::::::::::::::::::::::::::::::::::::::::::                 ::
at0210 CACCTCACCGGCATACGAATAAACTACGCTGGGGCGCGTTCATCTTTGTGTCGATCTCTGTTGTGTTCTTTCCCCTTCATAGCCGCACACACACACACACACACACACACACACACACCT

28IGR  GCC-GTTACAGTGCACTGCACGCACTTCCCGA-GCTCCCCACTTTATTTCTTCTCCACGCGCAAAGAGGCACGCTGCTGTGCATCGCGTGCGTGTGTGCGATCGTCTCTCTTCGCCGCTT
       ::: : :: : ::::::::  :          : :  :::::::::::::::::::::::: :::::::: : :: ::::   ::::  : :
at0210 GCCCGATAAAATGCACTGCGGGGGGGGGGGAAAGAACCCCACTTTATTTCTTCTCCACGCACAAAGAGGAAAGCAGCTGAACATCAGGAG-----------------------------

28IGR  TAGACCTCCTTGTGCATACCTCTCTCTGCGTGTGTCTTTCCTTTGTTTCTCCCCTAAAGT  Chromosome 28 (952.251 – 953.190)

at0210 ------------------------------------------------------------  Chromosome 13 (73.833 – 74.816)
```

Additional Figure S1. (A)The 3´ end of LbrM.13.0210 gene is derived from chromosome 28. In the intercoding region of LbrM.28.2580 and LbrM.28.2590 entries at chromosome 28 from *L. braziliensis* there is a 457 nucleotide sequence that is identical to the final 355 nucleotides of the LbrM.13.0210 ORF and the first 102 downstream nucleotides. Boxes in red comprise ORFs**. (B) Alignment of the LbrM.13.0210 entry with the intergenic region between the LbrM.28.2580 and the LbrM.28.2590 entries (28IGR)**. A 100% of identity in 457 nt overlapped, showed in red, was found. The ATG and stop codons of the LbrM.13.0210 entry are indicated in blue.
